# Supported Employment for the Reintegration of Disability Pensioners with Mental Illnesses: A Randomized Controlled Trial

**DOI:** 10.3389/fpubh.2015.00237

**Published:** 2015-10-20

**Authors:** Sandra Viering, Matthias Jäger, Bettina Bärtsch, Carlos Nordt, Wulf Rössler, Ingeborg Warnke, Wolfram Kawohl

**Affiliations:** ^1^Department of Psychiatry, Psychotherapy and Psychosomatics, Centre for Social Psychiatry, University Hospital for Psychiatry Zurich, Zurich, Switzerland; ^2^University of Zurich, Zurich, Switzerland; ^3^Laboratory of Neuroscience, LIM 27, Institute of Psychiatry, University of São Paulo, São Paulo, Brazil

**Keywords:** supported employment, social security disability insurance, mental illness, individual placement and support

## Abstract

Work is beneficial for the recovery from mental illness. Although the approach of individual placement and support (IPS) has been shown to be effective in Europe, it has not yet been widely implemented in European health care systems. The aim of this randomized controlled trial was to assess the effectiveness of IPS for disability pensioners with mental illnesses new on disability benefits in Switzerland. In the study at hand, 250 participants were randomly assigned to either the control or the intervention group. The participants in the intervention group received job coaching according to IPS during 2 years. The control group received no structured support. Both groups were interviewed at baseline and followed up every 6 months (baseline, 6, 12, 16, 18, 24 months) for 2 years. Primary outcome was to obtain a job in the competitive employment. IPS was more effective for the reintegration into the competitive employment market for disability pensioners than the control condition. Thirty-two percent of the participants of the intervention group and 12% of the control group obtained new jobs in the competitive employment. IPS is also effective for the reintegration into competitive employment of people with mental illness receiving disability pensions.

## Introduction

The number of people with mental illness actually working in the competitive employment market constitutes only about 10–20% (1). However, people with mental disorders wish to work in the competitive employment market. Furthermore, paid work is acknowledged as beneficial for recovery (2) against stigma (3), self-esteem, quality of life (4), and suicide prevention (5).

To reintegrate people with mental illness into competitive employment, two different vocational rehabilitation approaches exist. The first one, pre-vocational rehabilitation (PVR), has a long tradition in psychiatric rehabilitation and is based on the principle “first train then place.” This includes the training of skills and competencies relevant for employment delivered mainly in sheltered workplaces. Those services are firmly anchored in German-speaking areas (6, 7). The second approach, supported employment (SE), relies on the principle “first place then train.” This implies an integration into the competitive employment market in the first place with continuous support by a job coach, but without any preparatory training in a protected environment. In 1994, Becker and Drake (8) defined a specific SE approach for people with mental illness, which was supplemented by Drake et al. (9). This approach is called individual placement and support (IPS) and it is considered the best defined SE-method. IPS is based on eight principles: (a) competitive employment is the goal, (b) focus on individuals’ preferences, (c) welfare benefit counseling, (d) work closely with other care systems, (e) rapid job search, (f) individualized support, (g) time unlimited follow on support (also when the individual loses a job), and (h) the job coach needs to build up a network with potential future employers. In the past years, studies showed that IPS leads to improved competitive employment rates among individuals with mental illness compared to PVR (10–12). The effectiveness of the IPS approach has been well studied especially within the context of the US labor market (10). Furthermore, there is strong evidence that the IPS approach is effective in Europe, despite considerable variabilities in healthcare and social security systems compared to the US (13, 14). Although SE produces better employment outcomes and is more cost effective than PVR (15), it has not been widely implemented in European health care systems (16). This also accounts for Switzerland.

People suffering from mental illness frequently lose their jobs due to permanent disabilities caused by the illness (17). In Switzerland, it is possible to receive a full or partial pension when a disabling mental disorder can be verified. This means that if a person is considered fully incapacitated to work, a full pension will be payed. If a person is still able to work part time, he or she will receive a partial pension. Until 2010, the Swiss Federal Social Insurance Office (Bundesamt für Sozialversicherungen, BSV) registered a constant increase of people receiving disability pensions due to mental disorders (18). Since then this number of disability pensioners has been constant. The usual procedure in Switzerland is that once a person receives a disability pension the further rehabilitative support from the social insurance agency is limited, i.e., there are no structured efforts to reintegrate pensioners back into the competitive employment market. This can potentially lead to permanent unemployment and the mental disorder may become chronic (19). Early IPS for disability persioners may be a solution for this problem (20). The aim of this trial was to assess the effectiveness of IPS for pensioners due to mental illness at an early stage. Furthermore, we investigated the impact of IPS on secondary vocational outcomes.

## Materials and Methods

### Study Design and Participants

**Figure 1:**
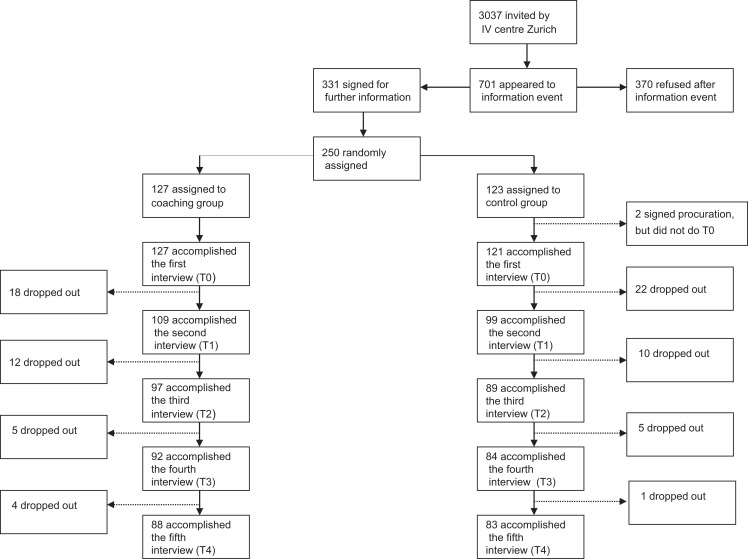
**Flow chart of recruitment process**.

Zürcher Eingliederungs Pilot Projekt [ZhEPP, eng.: Zurich integration pilot project; ISRCTN54951166; (21)] was carried out as a randomized controlled trial (RCT) at the University Hospital of Psychiatry Zurich (PUK). The study was conducted between January 2011 and September 2014. Possible participants qualified for enrollment if they lived in the canton of Zurich and received a disability pension (full or partial) due to mental illness for no longer than 1 year. The participants needed to be aged 18 years of age or older, wish to enter the competitive employment market or to remain there if they already had a job. Furthermore, all participants had to be in psychiatric and/or psychotherapeutical treatment during the whole study period. Mental retardation (diagnosed as ICD-10: F7) and organic mental disorder (diagnosed as ICD-10: F0) were exclusion criteria. A target sample size of 250 people was aspired. After having given informed consent the participants were randomly assigned to either IPS or the control group (see Figure 1). For the purpose of randomization, a list of numbers was created based on a Bernoulli distribution, a form of binomial probability distribution. Each participant was randomized according to that list.

During the following study period of 2 years, all participants were interviewed five times (at baseline, after 6, 12, 18, and 24 months) by research assistants. It was hypothesized that disability pensioners supported by IPS could be reintegrated more often into the competitive employment market than people who received the usual procedure applied in Switzerland and no additional support. Second, according to past research (9), we hypothesized that disability pensioners supported through IPS would reach higher income, work more hours and gain longer job tenure compared to the participants of the control group.

The ZhEPP study was funded by a grant from the Federal Social Insurance Office (BSV). The funding source had no influence on the design and the implementation of the study. The funding was not used to amplify the individuals’ income. The trial was conducted in accordance with the principles of good clinical practice and with the Declaration of Helsinki and its later amendments. The study was approved by the Ethics Committee of the Canton of Zurich (KEK-ZH-NR: 2010-0311/0). The corresponding author had full access to all the data in the study and had final responsibility for the decision to submit for publication.

### Intervention and Control Conditions

The intervention relied on the SE approach IPS. In total, there were four job coaches enrolled. Two of them were full-time employed, the other part time. All of them had a degree in psychology. The coaching frequency and the coaching duration of each session were determined individually by the job coach and the individual. No training of abilities or social skills neither any assessments of skills were administered beforehand. The job coach gave support during the application procedure (e.g., establishing realistic goals, writing applications, preparation of the job interview), and continued providing support according to the IPS principles during the participant employment (e.g., how to cope with workplace stressors including interpersonal conflicts with colleagues). The support was continued also in cases of job loss. Participants of the control group were free to choose for other vocational services including PVR, but were not supported by a job coach of ZhEPP. The primary outcome of the study was for the participants to obtain a job in competitive employment. We accepted the primary outcome as fulfilled if the job was obtained by standard application procedure (written application, CV, and job interview) and if the job was kept for at least 1 month. Secondary outcome parameters were the average number of hours and months worked, the number of months employed, and job tenure of the longest held job by the participant during the study period.

### Procedure and Materials

Participants were followed up for 24 months after the first interview. Data concerning socio-demographic characteristics, vocational outcome, hours worked, month employed, and job tenure were gathered using a structured questionnaire. All questionnaires were administered every 6 months. In the IPS group, job status was assessed every time a participant obtained a new job. The IPS fidelity scale was administered every 3 months. This 15 item-scale is a well-researched tool that evaluates the compliance of the service to the IPS principles as described in the introduction (22). High fidelity of the approach was stated if a job coach reached a score between 66 and 76 and moderate fidelity was rated for scores between 56 and 66. If the score of a job coach is <55, the fidelity of IPS services is considered insufficient (23). Participants received expenditure compensation of about 60 CHF (i.e., 40£) per interview (paid by parts of the funding of the BSV). Participants’ psychiatric diagnoses were gathered from the files of the IV-institution Zurich. All diagnoses were based on the International Classification of Diseases (ICD-10) and had been diagnosed by medical doctors.

### Statistics

For the general analyses, a sample size of 250 persons had been calculated using power analysis software G*Power (24). A medium effect size (0.42 SD) should be detected with a power of 95% at a two-tailed significance level of 0.05. All statistical analyses were conducted with SPSS 20.0. Data of all participants were analyzed as intention-to-treat. For that, the last observation carried forward (LOCF) method was used, meaning that in case of dropout, the last observation of a participant was used to replace the missing value. As some concerns exists that the use of LOCF can lead to an overestimation (or underestimation) of the effects (25), we additionally performed analyses only with the study completers (i.e., drop outs were rated as missing values and only those participants who participated until the end of the study were rated). With this analysis, we intended to control for the stability of the results.

Patient characteristics were analyzed descriptively (means, standard deviations, frequencies, and percentages). To test for normal distribution of continuous variables, the Kolmogorov–Smirnov-test was applied. Mann–Whitney-*U*-tests were performed if the variables were not normally distributed. Categorical variables were analyzed using cross tabulations with chi-square tests.

To test for the primary outcome, cross tabulations and chi-square tests were conducted. To test for group differences with respect of having competitive employment (i.e., being employed in the competitive employment market yes/no) over all measurements point, a generalized estimating equation model (GEE) was conducted. GEE is an advantageous model for the analysis of repeated measurements of categorical outcome variables. GEE was squared to allow more flexibility in handling possible fluctuations regarding the primary outcome during different measurement points. The development of the participants’ work in competitive employment across the study period is shown as a line graph. The differences between the single measurement points were analyzed by chi-square tests. The analyses of the secondary outcome variables were restricted to people who worked in competitive employment market for at least 1 month. The continuous secondary outcome variables: time of longest job tenure, hours worked per month, and number of months employed were analyzed with respect to group differences using Mann–Whitney-*U*-tests.

## Results

The overall drop-out-rate was 32% (79 participants, see Figure 1). The dropout rates in both groups were similar. Regarding baseline data, no significant differences between the IPS group and the control group were found (Table 1). Primary vocational outcome variables are outlined in Table 2. The primary outcome, (i.e., obtaining of a competitive employment yes/no) was scored as successfully fulfilled, if the job was kept for at least 1 month. The first item in Table 2 (i.e., “Total numbers of jobs obtained”) summed multiple jobs of a single participant, if applicable.

**Table 1:** **Patient characteristics at baseline**.

	IPS (*n* **=** 127)	Control group (*n* **=** 123)	Total (*n* **=** 250)
Age	41.7 (10.3)	43.7 (10.8)	42.6 (10.6)
Woman	69 (54%)	63 (53%)	132 (53%)
Age at first psychiatric contact (years)	31.07 (12.2)	33.56 (11.8)	32.26 (12.1)
Number of admissions in lifetime			
0	42 (33%)	33 (24%)	75 (30%)
1–5	74 (58%)	71 (58%)	145 (58%)
6–10	7 (6%)	12 (10%)	19 (8%)
11+	3 (2%)	3 (2%)	6 (2%)
Clinical diagnosis			
Mood affective disorder	60 (47%)	58 (47%)	118 (47%)
Schizophrenia/schizoaffective disorder	21 (17%)	18 (15%)	39 (16%)
Personality disorder	22 (17%)	21 (17%)	43 (17%)
Other	23 (18%)	22 (18%)	45 (18%)
Unemployed at baseline work history	92 (72%)	91 (74%)	183 (73%)
>1 month in past years	69 (54%)	57 (47%)	126 (50%)
<1 month in past year	54 (43%)	64 (52%)	118 (47%)
Number of years in school education graduation	10.2 (1.6)	10.1 (1.8)	10.2 (1.7)
Primary school	3 (2%)	3 (2%)	6 (2%)
Basic school (9 years)	88 (70%)	80 (65%)	168 (67%)
Abitur (high-school)	17 (13%)	20 (16%)	37 (15%)
Other	17 (13%)	16 (13%)	33 (13%)
Living situation			
Alone	68 (54%)	64 (52%)	132 (53%)
With friends/relatives	46 (36%)	45 (37%)	91 (36%)
Other	13 (10%)	10 (8%)	13 (5%)
Born in country of residence	104 (82%)	91 (74%)	195 (78%)

Data are mean (SD) or number (%). Some baseline characteristics were missing, since not all patients did supply this information. *p* > 0.05

**Table 2:** **Employment in the yourse of the study**.

	LOCF			Study completers		
	IPS (*n* = 127)	Control group (*n* = 121)	*p*	IPS (*n* = 88)	Control group (*n* = 85)	*p*
Number of new jobs obtained	40 (32%)	14 (12%)	<0.0001***	40 (46%)	14 (16%)	<0.0001***
Number of participants with no job at baseline but in the end of study	22 (17%)	10 (8%)	0.049*	19 (22%)	9 (11%)^a^	0.046*
Number of participants without any job during the study	64 (50%)	80 (66%)	0.012*	35 (40%)	54 (64%)^b^	0.002**
Number of participants with continuous employment	27 (21%)	21 (17%)	0.437	19 (22%)	15 (18%)^c^	0.490

Date are number (%), *p < 0.05; **p < 0.01; ***p < 0.0001.

*The group of study completers contains results regarding solely participants who participate until the study end. Amout of participants who were recorded as drop out: ^a^IPS = 3, control group = 1; ^b^IPS = 29, control group = 26; ^c^IPS = 8, control group = 6*.

**Figure 2:**
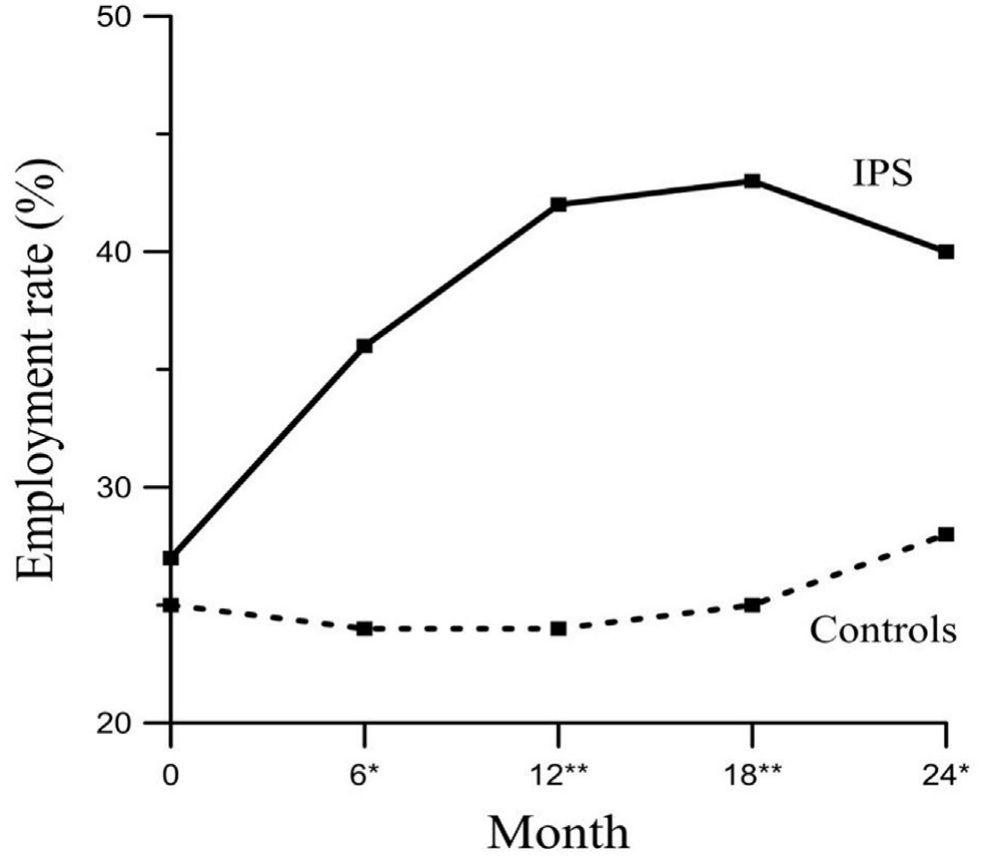
**Employment rates of competitive employment for IPS and control group throughout the whole 2-year period (LOCF) including jobs that had been held already at the beginning of the study**. Chi-Quadrat-test; *p < 0.05; **p < 0.01. The total numbers of jobs during each time of measurement.

Figure 2 shows the development of both groups regarding employment rates using LOCF. In total, 17 participants dropped out, from the participants who had a job in the beginning, eight participants of the coaching group, nine from the control group. The GEE method revealed significant interactions between the covariates time and group indicating a significant difference between the groups over time. Therefore, for each group (IPS vs. control group) add 0.288 to the intercept (0.212; *p* < 0.05). It can be seen that after 6 months the groups differ by 0.113 (Table 3).

**Table 3:** **Model estimates the amount of job regarding different measurement points**.

	Estimate	SE	df	Model fit
Intercept	−1.109	0.212***	1	1452.9
Group	0.103	0.288		
time	−0.080	0.113		
time^2^	0.031	0.025		
Group* time	0.599	0.164***		
Group* time^2^	−0.122	0.036**		

Group = contains IPS and control group, time = contains the five different measurement points, Model fit = time squared (curvilinier time trend). *p < 0.05;**p < 0.01; ***p < 0.001.

Table 4 depicts group differences concerning the secondary (continuous) vocational variables. The variables assessed included hours and months being employed, and job tenure during the whole study process. There was no significant group difference for any of these variable for both analyses (LOCF and Study completers). These results could be replicated by using the study completers only.

**Table 4:** **Results of secondary outcome variables**.

	LOCF			Study completers		
	IPS (*n* = 63)	Control group (*n* = 41)	*p*	IPS (*n* = 51)	Control group (*n* = 31)	*p*
Average month employed	41.70 (70.20)	42.94 (73.91)	0.244	34.9 (61.5)	44.3 (79.6)	0.209
Average hours worked per month	47.37 (30.33)	44.37 (31.19)	0.552	48.6 (30.99)	41.43 (29.6)	0.263
Job tenure of the longest job held	51.25 (70.63)	57.85 (81.31)	0.503	43.7 (63.3)	53.1 (76.1)	0.363

*The table includes jobs that had been held already at the beginning of the study*.

*Group has been reduced on participants who worked in competitive employment market. Data were presented as mean (SD), Mann–Whitney-*U*-test*.

Moderate IPS fidelity was given throughout the whole study period (*M* = 61.2, SD = 3.03). Most items had high scores (min–max 3.8–5). However, two items, item 4 (“cooperation with other institutions and other care team individuals”) and 14 (“community-oriented services”), were rated low.

## Discussion

The results of our study support the assumption that IPS is effective in the reintegration of people with mental illnesses into the competitive employment market of Europe (26). Regarding the primary outcome (i.e., being employed in the competitive employment market for at least 1 month), it has been shown that pensioners with mental illnesses supported by IPS obtained significantly more new jobs in the competitive employment market than participants of the control group. These findings are consistent with the EQOLISE study and reveal that it is useful to reintegrate disability pensioners at an early stage using IPS. However, the time criterion chosen was more conservative as in the EQOLISE study, in which a criterion of employment for at least one day was applied (13). This inures to the benefits of common goals of psychiatric rehabilitation, i.e., participation in society, protection against social isolation (27) and reduction of the risk that a mental illness becomes chronic (19). Furthermore, these findings were consisten with the results of the study by Drake et al. (28). This study included Social Security Disability Insurance (SSDI) beneficiary in the US and found that the beneficiaries who received support through SE obtained more often competitive employment (52.4%) compared to the control group (33.0%) who received the standard procedure. However, this study lacks a time specification describing since when the beneferiaries received the SSDI.

Regarding the current study, the number of reintegrated participants in the IPS group increased initially, but declined slightly after 18 month while the reintegration rate of the control group increased continuously but less considerably. To the best of our knowledge, this has not been observed in the majority of previous studies. However, in a catamnestic survey of the participants of the EQOLISE study in Zurich a similar effect has been found (29). One explanation could be that other studies had shorter observation periods up to a maximum of 18 months (13, 30). However, Hoffmann et al. (31) stated that even the period of 24 month applied in their study was possibly too short to investigate the sustainability of IPS. Due to the follow-up of this study, it could be shown that the suistainibility is given over a 5-year study period (32). Another explanation for the decrease of the effect after 18 months in our study might be a spill-over-effect. This effect means that participants of the control group might be orientated toward the intervention group (33), e.g., by being interviewed and thus being in touch with the subject of vocational rehabilitation. Further explanation could be the effect of time, meaning a rising probability of finding a job even without any support.

As this study did not find any significant differences between IPS and control group regarding hours and months worked as well as job tenure. Therefore, the assumption that IPS leads to higher income and more time being employed at work (34) could not be supported. However, our study stands out because it includes people with mental illnesses, who already had a job at the beginning of the study and thus earned additional salaries. However, previous studies have shown that work is beneficial for recovery, not just because of financial aspects, but also to feel needed and to build a social identity (35). Even more important, in a worldwide survey Nordt et al. (4) showed that unemployment is related to a 20–30% increase of the relative suicide risk. Thus effects associated with unemployment should also be targeted in the context of suicide (36).

Some mental health professionals believe that going back to employment may worsen the mental health condition of their patients (37). Especially stressful surroundings, common in a competitive employment market, are seen as a major risk factor to people with mental illnesses (35). In contrast to the apprehension of the mental health professionals, previous research showed that people with mental illnesses stated that they wanted to work in competitive employment market (38). Furthermore, based on our moderate dropout rate of about 30% and the fact that most participants dropped out during the first 6 months, we conclude that the participants who participated until the end were motivated to be reintegrated into competitive employment market.

Our study is not without limitations. Usually, high IPS fidelity leads to high effectiveness of IPS (10, 39). The results of this study regarding IPS fidelity are not fully satisfactory. Especially two items of the IPS fidelity scale, cooperation with other institutions and other care team individuals and community-orientated services, did not meet a sufficient level. In addition, in this study not the latest version of IPS fidelity scale was used (40). The fidelity study was cenceptualized in 2009 and started in January 2011, the latest version was validated only in 2012.

Furthermore, to increase the knowledge about predictors influencing the effectiveness of IPS, future publications should be focused on that topic. However, the cost efficacy in Switzerland regarding IPS as a standard service has not been investigated yet. This should also be a focus of future research.

In conclusion, this study shows that mentally ill, disabled pensioners recently on social benefits in Switzerland can profit from IPS. As the result of the ZhEPP-trial supports the general finding of the effectiveness of IPS in Switzerland (13), it is indicated that IPS should be included as a standard service in vocational rehabilitation, also at an early stage of retirement.

## Author Contributions

WK designed the study and served as the principal investigator. SV and CN did the statistical analysis. SV, MJ, and WK drafted the manuscript. IW contributed to statistical analyses and revised the final manuscript. All authors participated considerably in writing of this manuscript.

## Financial Support

This study was funded by the Swiss Social Insurance Office (BSV). This funding was used for the salary of the job coaches and the scientific personell and for the compensation of the interviews. No money was used to amplify parcticipants’ income.

## Conflict of Interest Statement

Wolfram Kawohl is the head of the Center for Social Psychiatry of the PUK. In this role, he is the supervisor of an IPS unit. Moreover, he is a member of the board of a vocational rehabilitation institution (PVR). The authors Sandra Viering, Matthias Jäger, Bettina Bärtsch, Carlos Nordt, Wulf Rössler, and Ingeborg Warnke declare that the research was conducted in the absence of any commercial or financial relationships that could be construed as a potential conflict of interest. The Associate Editor Alexandre Loch declares that, despite having collaborated with author Wulf Rössler, the review process was handled objectively and no conflict of interest exists.
